# Quadcopters Testing Platform for Educational Environments

**DOI:** 10.3390/s21124134

**Published:** 2021-06-16

**Authors:** Uriel Veyna, Sergio Garcia-Nieto, Raul Simarro, Jose Vicente Salcedo

**Affiliations:** Instituto Universitario de Automática e Informática Industrial, Universitat Politècnica de València, 46022 Valencia, Spain; urveyro1@doctor.upv.es (U.V.); rausifer@upv.es (R.S.); jsalcedo@upv.es (J.V.S.)

**Keywords:** design, construction, gyroscopic structure, quadcopter, state feedback, PID, Matlab-Simulink, experimental prototype

## Abstract

This work focuses on the design and construction of an experimental test bench of three degrees of freedom with application in educational environments. It is constituted by a gyroscopic structure that allows the movements of a quadcopter to analyze the control systems. In this context, the main features of the mechanical and electronic design of this prototype are described. At the same time, the main characteristics with respect to existing platforms are highlighted in aspects such as: system autonomy, cost, safety level, operation ranges, experimental flexibility, among others. The possible controller design approaches for quadcopter stabilization can extend to many basic and advanced techniques. In this work, to show the operation and didactic use of the platform, the development of the controller for tilt angle stabilization under two different approaches are presented. The first approach is through PID control, oriented for undergraduate students with basic level in control theory. The second approach is by means of State Feedback, oriented to students with more advanced level in this field. The result of this work is an open test bench, enabled for the experimentation of control algorithms using Matlab-Simulink.

## 1. Introduction

Recently, unmanned aerial vehicles (UAVs) have shown a remarkable advance in their technological development. Specifically, vertical take-off and landing (VTOL) aircraft are of special interest since they do not need a runway to perform take-off or landing manoeuvres [1]. There are several configurations for this type of aircraft and one of the most widely used is the quadcopter, whose flight stability is defined by regulation of revolution speed between its four motors. This type of UAV are widely used as experimental platforms due to their mechanical simplicity and inherent robustness [2]. Trends imply that analyses on quadcopters are becoming more and more relevant in the field of aerial robotics, as they are considered very practical prototypes in university teaching to promote programming and robotics skills [3]. However, educational platforms based on multirotors are very challenging in terms of design and control due to their nature of operation, as they require moving in a three-dimensional space.

The design of an attitude control algorithm for a quadcopter requires a long period of development including analysis under simulation and flight testing. Most control systems require an experimental phase on the physical system for validation, since unexpected performances may occur as a consequence of limitations in the control design, e.g., unmodelled dynamics [4]. For this reason, there is a necessity to use test platforms that allow an easier transition between numerical and experimental analysis, by evaluating the performance of a controller under a safe environment for user and vehicle, without any risk of collision [5].

From a teaching perspective of control systems involved with UAVs, some contributions presented are the software platform [6], in which detection, tracking and control algorithms can be evaluated and tested all together in a 3D graphical tool. There are also educational multi-rotor platforms such as [7], with intuitive software that allows the basic learning of aerodynamic principles. Hardware-in-the-loop (HIL) platforms represent an effective approach in UAV design due to their potential to execute simulations and experiments [8]. In university laboratories, it is also common to find the use of different types of inverse pendulums for teaching control systems [9].

All these platforms represent experimental tools that allow students to evaluate and tune control algorithms, which operate based on the application of inertial devices such as micro-gyroscopes that measure the angular velocities and positions of the system. Nowadays, the analysis of control systems based on this technology is highly important in control engineering subjects, since it has many automotive applications, such as anti-rollover systems, antiskid control and electronic stability control. Some consumer applications are camera stabilization, cell phone stabilization, inertial mouse and navigation for portable electronics. In addition, micro-gyroscopes have a wide range of military applications on missiles navigation, aeronautics and astronautics, stable platform, GPS and so on [10].

This article presents the design and construction of a three degree of freedom (DoF) experimental platform for validation of control systems before being implemented in quadcopters. Likewise, its design is oriented to be used for teaching purposes in university laboratories. Regarding the development of control systems in aerial vehicles with four rotors, some of the different control techniques that are used for research and teaching are proportional integral derivative (PID) control, linear quadratic regulator (LQR) [11] and linearization by feedback [12,13]. Other kinds of more advanced control strategies are also mentioned in such as H infinity [14], fuzzy logic [15], back-stepping [12,16], dynamic inversion [17], sliding mode (SMC) [18] and so on.

The outstanding features of the proposed platform in comparison with existing prototypes involve the following aspects: energy (system autonomy), programming level, cost, safety level, system operation ranges, experimental flexibility and replication level. These highlights contribute to a better adaptation of the proposed prototype to the student environment for performing laboratory sessions.

The academic perspective of the testing platform is to allow students to evaluate the approach of a control system experimentally, partially or totally, modifying the control algorithm through the editing of Matlab/Simulink block diagrams.

To show the potential of this prototype in an educational environment, this work considers laboratory tasks focused on the design of controllers for two different groups of undergraduate students, as described below:

Student group A: Students belonging to basic level control and automation courses, with lacking base knowledge and no notions of implementation in real physical systems.

Student group B: Students belonging to intermediate level control and automation courses, with solid basic knowledge and without notions of implementation in real physical systems.

As a global approach, for the group of students A, the development of a classical PID controller is proposed. While for the group of students B, the development of a controller using the state feedback technique is proposed. For both cases, the working hypothesis is considered a basic knowledge in control theory from the student’s side, so basic control schemes are used, in continuous time, obviating implementation issues, as the discretization of the controller or the use of the filtered derivative term. Despite this, it should be noted that it is possible to use the platform for the evaluation of more sophisticated control designs that consider these aspects, typical of courses for more advanced graduate students, master’s degree, etc.

The remainder of the paper is organised as follows. Section 2 provides a concise overview of the prototype developed in this work. Section 3 presents background about prototypes with similar approaches. Section 4 discusses the kinematics and dynamics principles of the prototype. Section 5 describes the methods, materials and components associated with both the electronic and the structural design of the prototype. Section 6 describes the hardware and software implemented in the project. Section 7 describes the system parameter identification. In Section 8, the model and strategy for designing the controller to be implemented in the system are formulated. Section 9 shows the development of the control laws to be evaluated in the platform. Section 10 shows the implementation process and the numerical and experimental results obtained. Section 11 summarizes the conclusions.

## 2. System Overview

**Figure 1:**
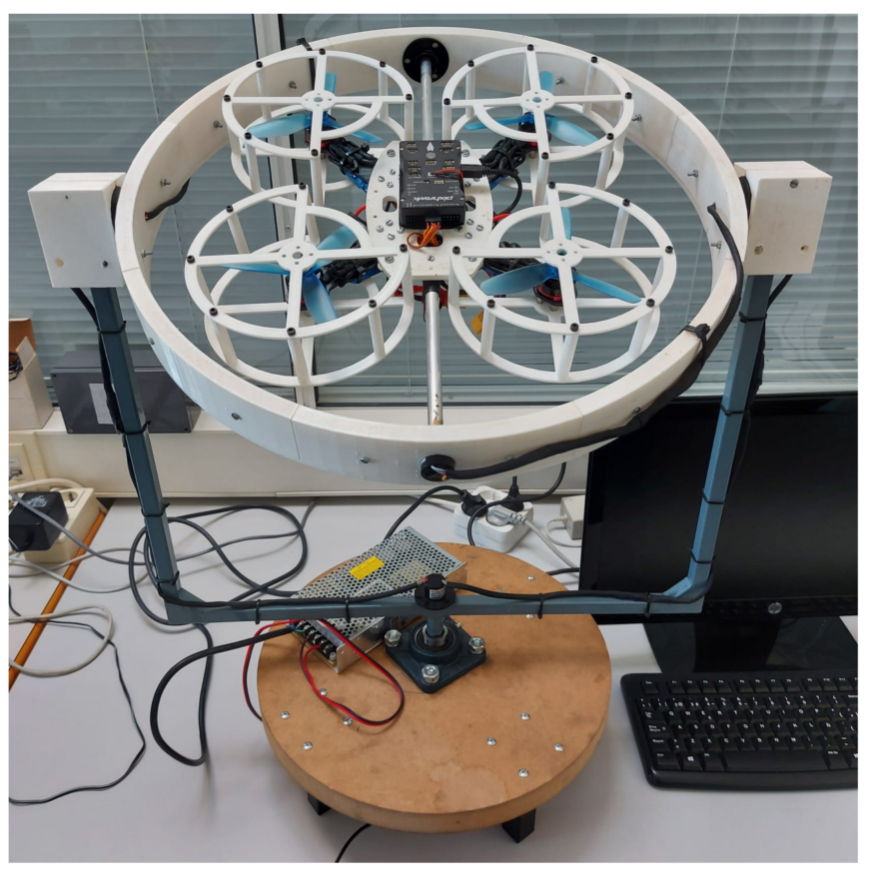
Experimental prototype of three degrees of freedom for validation of control algorithms in quadcopter systems.

The prototype built for this work and previously mentioned is presented in Figure 1, it performs the function of a test bench for control law evaluation designed for the stabilisation of aerial vehicles with four rotors. It is constituted by a system of 4 main elements, which are: a gyroscopic structure with three degrees of freedom, a quadcopter, an external power supply and a computer. Each of these elements is described in more detail below.

- Three degrees of freedom gyroscopic structure: mechanical structure that enables pitch, roll and yaw motions in a quadcopter, while at the same time constraining translational movements.
- Quadcopter: aerial vehicle with specifications for control law experimentation inside a laboratory.
- External power supply: element responsible for supplying electric current to the quadcopter.
- Computer: auxiliary tool for communication between user and platform in implementation of control algorithms using Matlab-Simulink.
  Some technical specifications of the test platform are presented below.
- Dimensional specifications
  Maximum volume: 0.1235 m^3^ ; base diameter: 0.38 m ; total weight: 2.93 kg ; maximum height: 0.56 m
- Motion specifications
  Allowed ranges of motion: 360 degrees for roll, 360 degrees for pitch and 0 degrees for yaw.
- Power specifications
  Propellers used: 10.14 cm length, 11.43 cm pitch, three blades. Motor revolution constant: 2300 kv; maximum power supply rating: +12 V; 12.5 A.

Since this platform is designed to be used as an educational tool in robotics and automation laboratories from basic courses, the prototype incorporates two powerful IMUs combined with a sensor fusion algorithm that allows obtaining measurement signals with very low noise levels. Consequently, students in introductory control courses can focus on the design of basic control strategies without the need to consider implementation details. However, it is possible to formulate more sophisticated strategies for advanced courses, where the use of observers, filters, predictors, etc. can be considered. As an example of an application, a video of this prototype in operation can be found at [19].

In introductory courses, the main objective for the students is to be able to verify, evaluate and experiment with the parameters/gains of the control system, which is predetermined by the teacher. On the other hand, in advanced courses, the selection and implementation of the control structure can be considered. Some of the tasks proposed for the students are the following:

**Possible control systems laboratory assignments**

- Design of a controller for angular stabilization
- Design of a controller for angular velocity
- Design of a controller for disturbance rejection
- Design of an uncertainty tolerant controller
- Design of a fault tolerant controller
- Verification of mathematical models

**Possible robotics lab assignments**

- Identification of multirotor system elements
- Understanding of basic multirotor maneuvers
- Control and operation of brushless motors
- Application of inertial sensors
- Signal processing and tuning of noise filters

As in most laboratory activities, it is required that students have some prior theoretical knowledge in certain areas of engineering. According to the laboratory work to be performed in relation to the level of complexity of the student subject in the area of control theory, it is desirable that students possess certain prior knowledge. In the Table 1, the basic areas of knowledge desirable for students in the performance of laboratory tasks are indicated. Further, the areas of knowledge with preferably intermediate or advanced level that is desirable in students in relation to the complexity of the task to be performed with respect to the content of the current student subject are indicated. As is usual in university courses, an introductory theory lesson may be given before students are asked to perform a laboratory task.

## 3. Background and System Approach

Experimental tests under completely safe conditions are very important for the development of flight controllers. Since it is possible to find unconsidered variations in vehicle behaviour, the use of a test platform becomes an interesting solution [5].

The main goal of the prototype shown in Figure 1 is to minimize the difficulty of experimental testing for quadcopter systems in reduced spaces with appropriate safety measures. The design of this prototype is focused on academic applications, due to its dimensions and manufacturing method it is ideal to be installed and replicated in university laboratories where it can be used to analyse quadcopter dynamics under any control law proposed. In addition, with the auxiliary use of MATLAB-Simulink software for algorithm implementation and sensor monitoring, this prototype becomes more accessible to be used by engineering students.

The state of the art shows the existence of some mechanisms used as test benches for UAVs, some of them have been developed by research groups such as the Australian National University [20], Stanford University [21], the Swiss Federal Institute [22] and the University of La Rioja [23], this last prototype manages to solve some problems that other platforms have been facing, such as limitations in time of use, construction complexity, low level of reproducibility and design conditions such as lightness and balance of components concerning vehicle’s center of gravity. However, it lacks a protection system that would naturally increase the safety level.

**Figure 2:**
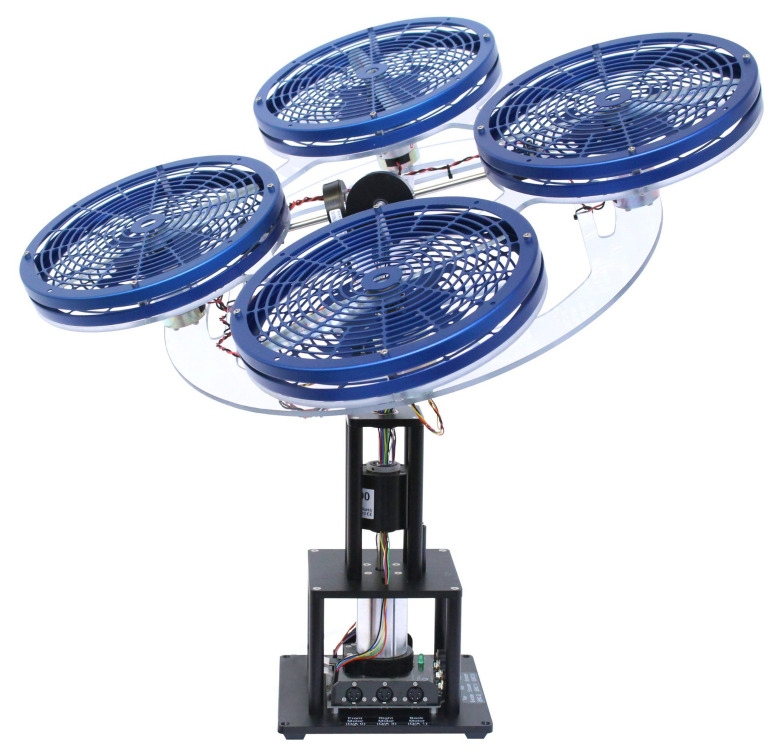
three DOF Hover prototype developed by Quanser.

Apart from these platforms, there are others available on the market, such as the FFT GYRO platform developed by Eureka dynamics or the three DOF Hover prototype shown in Figure 2 which belongs to Quanser [24]. This last platform consists of three degrees of freedom mechanism that is equipped with three decoders that provide information about the platform inclinations for their control and stabilisation. However, this mechanism adopts a configuration based on a fixed ball and socket joint that limits the platform rotation angles in a given range. Table 2 compares some features of the prototype presented in this article (PT-1), the platform developed by the University of La Rioja (PT-2) and the commercial platforms FFT GYRO (PT-3) and three DOF Hover (PT-4).

Some features that stand out in PT-1 with respect to the others are as follows:

- The power supply does not depend on the discharge time of a conventional UAV battery as in PT-3.
- It has full rotation range in its three axes unlike PT-4.
- It has a protection system that increases its safety level with respect to PT-2 and PT-3.
- The incorporation of MATLAB-Simulink for control algorithm programming promotes its adaptation to educational environments.
- It can adapt different quadcopter vehicles with similar geometrical dimensions, since they can be mounted on the gyroscopic structure without any difficulty. PT-2, PT-3 and PT-4 platforms do not have this feature since their design are more rigid.
- Due to the method for algorithm implementation that is proposed (external mode) and described later in Section 6.2, it is possible to define tuning parameters in the control algorithm that can be regulated during experimentation. This adds extra flexibility in the development phase of a control system.
- Since it is composed mostly of elements manufactured by 3D printing, its construction has a low level of complexity, which makes it feasible to replicate for laboratories and at lower cost compared with the market price of PT-3 and PT-4 platforms.

## 4. System Dynamics and Kinematics

As mentioned in [25], in previous work related to UAVs, mathematical models derived from the reference of aerial vehicles with similar dynamics have been used to reach quite identical conclusions. However, some assumptions made for other types of aircraft may not be appropriate for quadcopters due to the fact that they do not accurately represent the kinematics of this particular system. To design appropriate attitude controllers, the use of an accurate dynamic model is required. In [26] they emphasize the importance of using models based on capturing the existing dependence between the dynamics and kinematics of the system for obtaining governing equations of motion.

The following is a brief description of physical foundations of the prototype in Figure 1. The dynamic performance of a quadcopter and the kinematics of the gyroscopic structure are exhibited.

### 4.1. Quadcopter Dynamics

**Figure 3:**
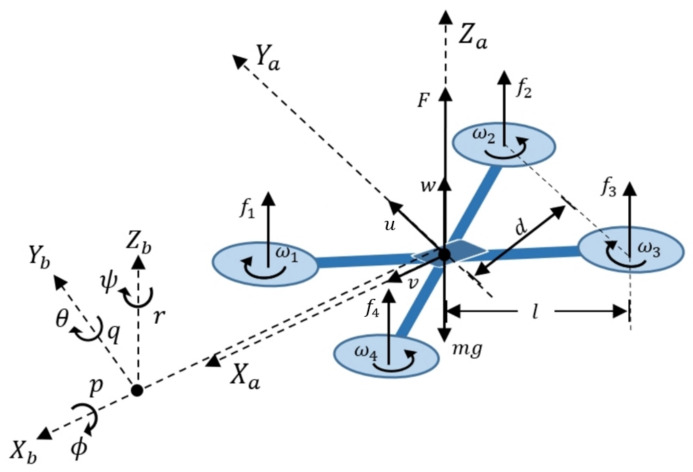
Free-body diagram of a quadcopter.

The six quadcopter degrees of freedom are defined by its rotational and translational motions, which are generated from forces and moments produced by each of its four motors [17]. The free-body diagram of a quadcopter is shown in Figure 3. The reference frame $\left[X_{a},Y_{a},Z_{a}\right]$ is fixed and used to describe translational displacements, while system $\left[X_{b},Y_{b},Z_{b}\right]$ represents the inertial reference frame which rotates with the vehicle. Each motor is located at a distance *l* from the center of the vehicle and runs at speed $\omega_{n}$ to generate a thrust force $f_{n}$ with perpendicular direction to the plane of rotation. The vector sum of all forces is the net force *F*, which acts at the center of mass and counteracts the total weight mg of the drone when it is hovering at a fixed point. Displacement through space at a linear velocity [u,v,w] is caused by variation of forces $f_{1},f_{2},f_{3}$ and $f_{4}$, which magnitudes are regulated by the rotational speed of each motor, and their directions by induction of angular moments on the reference frame $\left[X_{b},Y_{b},Z_{b}\right]$ [27]. These torques are generated from the application of [$\tau_{\phi },\tau_{\theta },\tau_{\psi }$] that give rise to rotational motions in the vehicle, which are known as pitch, roll, yaw and are defined by the euler angles [ϕ,θ,ψ]. The relationship between [$\dot{\phi },\dot{\theta },\dot{\psi }$] and angular velocities [p,q,r] is presented later in Section 8.1.

To generate clockwise and counterclockwise yaw torques, the configuration of motors is set such that velocities $\omega_{1}$ and $\omega_{3}$ have clockwise direction, while velocities $\omega_{2}$ and $\omega_{4}$ have counterclockwise direction as shown in Figure 3. With reference to system [$X_{a},Y_{a},Z_{a}$], translation of quadcopter is produced as a consequence of the forces and torques that are defined by Equations (1)–(5).

Displacement at $Z_{a}$ is caused by the effect of the vertical component of net force *F* (1) with respect to vehicle inclination.

(1) $$F=\sum_{i=1}^{4}f_{i}$$

Pitching motion (angular displacement θ) is created from torque $\tau_{\theta }$ (2), which rotates vehicle on $X_{b}$ axis and as a result it induces a translational motion on $Y_{a}$ axis.

(2) $$\tau_{\theta }=d\left(f_{1}-f_{2}-f_{3}+f_{4}\right)$$

where *d* is the geometric distance in perpendicular direction between the point of application for each force ($f_{n}$) and the axis of rotation, defined by the Equation (3).

(3) d=lcos(π/4)

Roll motion (angular displacement ϕ) is created from torque $\tau_{\phi }$ (4),which generates rotation on $Y_{b}$ axis, causing translation on $X_{a}$ axis.

(4) $$\tau_{\phi }=d\left(-f_{1}-f_{2}+f_{3}+f_{4}\right)$$

And finally, yaw motion (angular displacement ψ) is originated by the total sum of drag torques generated from each propeller (5), resulting in rotation on $Z_{b}$ axis.

(5) $$\tau_{\psi }=c\left(f_{1}+f_{3}-f_{2}-f_{4}\right)$$

where *c* is a positive yaw constant that depends on the ratio of thrust to drag of motors.

### 4.2. Kinematic Description of the Gyroscopic Structure

**Figure 4:**
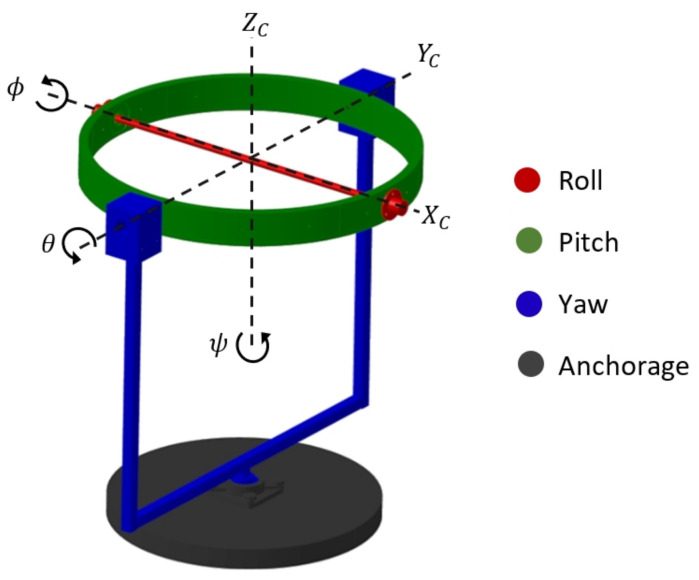
Kinematic description of the gyroscopic platform.

The design of this test platform is focused on the dynamic principles of a quadcopter to perform its angular motions. Figure 4 represents kinematic operation by describing the rotational motion of each platform component in relation to the reference system $[X_{C}$,$Y_{C}$,$Z_{C}]$.

Elements in red rotate on $X_{C}$ axis and have the function of enabling roll ϕ motion. Elements in green enable pitch motion θ rotating on $Y_{C}$ axis. Elements in blue define rotation on $Z_{C}$ axis establishing a yaw orientation ψ. Elements in black are anchors for the entire prototype.

## 5. Methods, Materials and Components

The following is a general description of the mechanical and electronic structure of elements that constitute the prototype.

### 5.1. Quadcopter Structural Design

**Figure 5:**
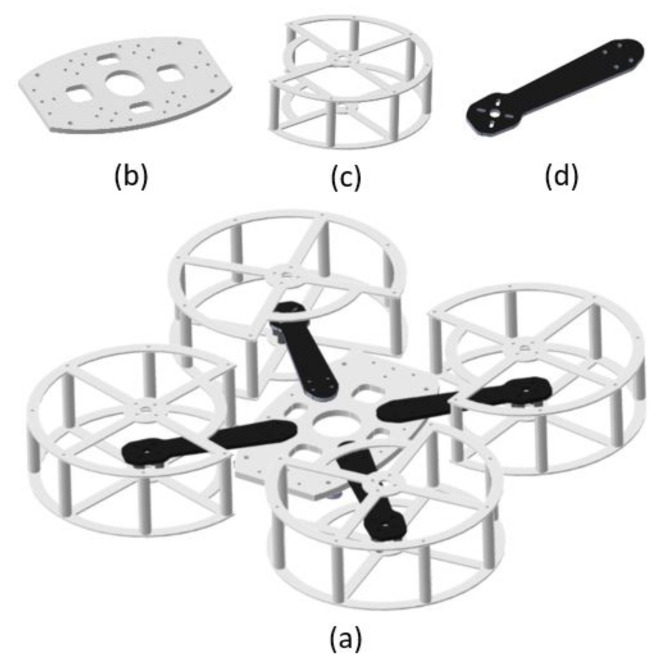
(**a**) CAD model of quadcopter structure assembly. (**b**) Center plate. (**c**) Propeller protector. (**d**) Arm.

The quadcopter structural system has two essential functions, to resist forces and torques present in its dynamic performance and to contain the necessary electronic components for its operation. The structure designed for this prototype (Figure 5a) is based on the architecture of a set of quadcopters with low commercial value on the market, which contributes to a higher level of replicability.

It has a symmetrical configuration, such that each motor has a location equidistant to the geometric center. It is designed to be light, resistant and has a set of protectors to protect the user’s integrity during the operation phase. Figure 5b–d show the three main components that constitute the vehicle structure, which are specified below.

- **Center plate:** Element that joins the four quadcopter arms. It is made of PLA material by 3D printing. Its geometry comes from an elliptical segment and has a thickness of 5 mm.
- **Arms:** Components responsible for supporting the motors. Made of carbon fibre with a thickness of 3 mm and length of 250 mm.
- **Propeller protectors:** Made of PLA material by 3D printing. They have a thickness of 2 mm, height of 50 mm and diameter of 160 mm.

The .cad files of the 3D printed parts of this structure can be found in the documentation folder for this prototype, which is accessible at [28].

### 5.2. Gyroscopic Platform Structural Design

The structural function of the test platform is to hold the quadcopter system so that its natural rotational motions are enabled. For this, slip rings [29] are used to transmit free rotational motion between pieces. In addition, these components are also used to conduct electrical current and USB communication signal through their connection channels. Flexibility in the design of this structure allows the coupling of different aerial vehicles with similar dimensions. This feature expands the scenario of possible experimentation analysis using this prototype.

**Figure 6:**
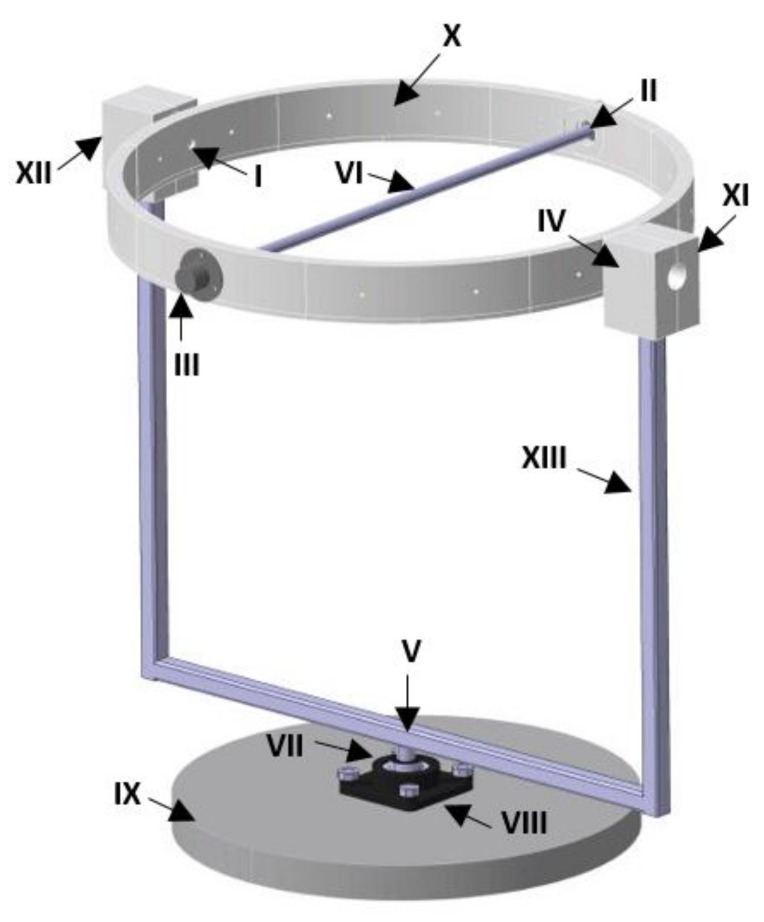
Elements of structural system that constitute gyroscopic platform.

The following is the description of each structural element that constitutes the test platform according to the nomenclature shown in Figure 6.

Roll movement is executed by the action of an aluminium tube (VI) which is connected between two slip rings located in II and III. At the same time, these slip rings are integrated into the main hoop (X) that is used to perform pitching motion. This hoop is made up of 16 pieces joined together, of which 8 of them have male configuration and 8 female configurations, each piece constitutes 45 degrees of the circumference that forms the complete hoop. From female pieces that form this hoop, 4 of them are modified in a different way for the adaptation of slip rings located in numbers I, II, III and IV. These modifications consist of a hole located in the center of each piece for insertion of the shafts of each slip ring. A metal frame with a square cross-section (XIII) is used to support the assembled hoop. Two rectangular supports (XI and XII) are attached to the upper ends of this frame to hold slip rings I and IV. The lower end of this frame is embedded through two supports (VII and VIII) to a circular wooden base (IX) which is used as an anchor point for the whole prototype.

As well, the .cad files of the components that integrate this platform and are 3D printed can be found in prototype documentation at [28].

### 5.3. Quadcopter Electronic Design

**Figure 7:**
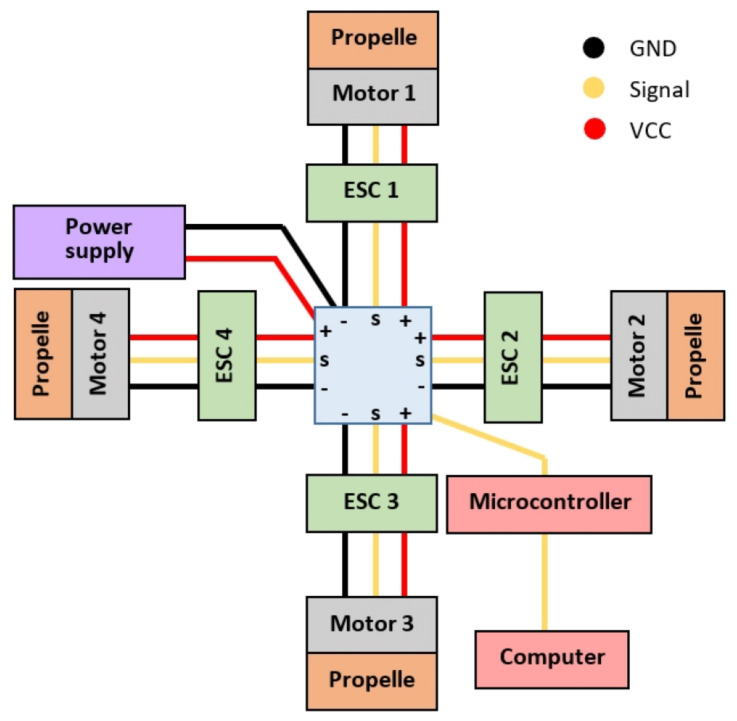
Quadcopter electrical connection diagram.

The quadcopter electronic system is integrated by devices specified in Table 3, which are connected according to the diagram shown in Figure 7. Yellow lines represent communication signal, while red and black lines represent VCC and GND poles respectively for transmission of electric current.

As one of the main criteria considered in the electronic design, component selection is based on the economic feasibility to build multiple replications of the prototype. In this sense, the selected set of electronic components is based on devices used in qav250 quadcopter model, since it is relatively inexpensive, it is widespread internationally and it is easy to find any necessary component for replacement.

### 5.4. Gyroscopic Platform Electronic Design

**Figure 8:**
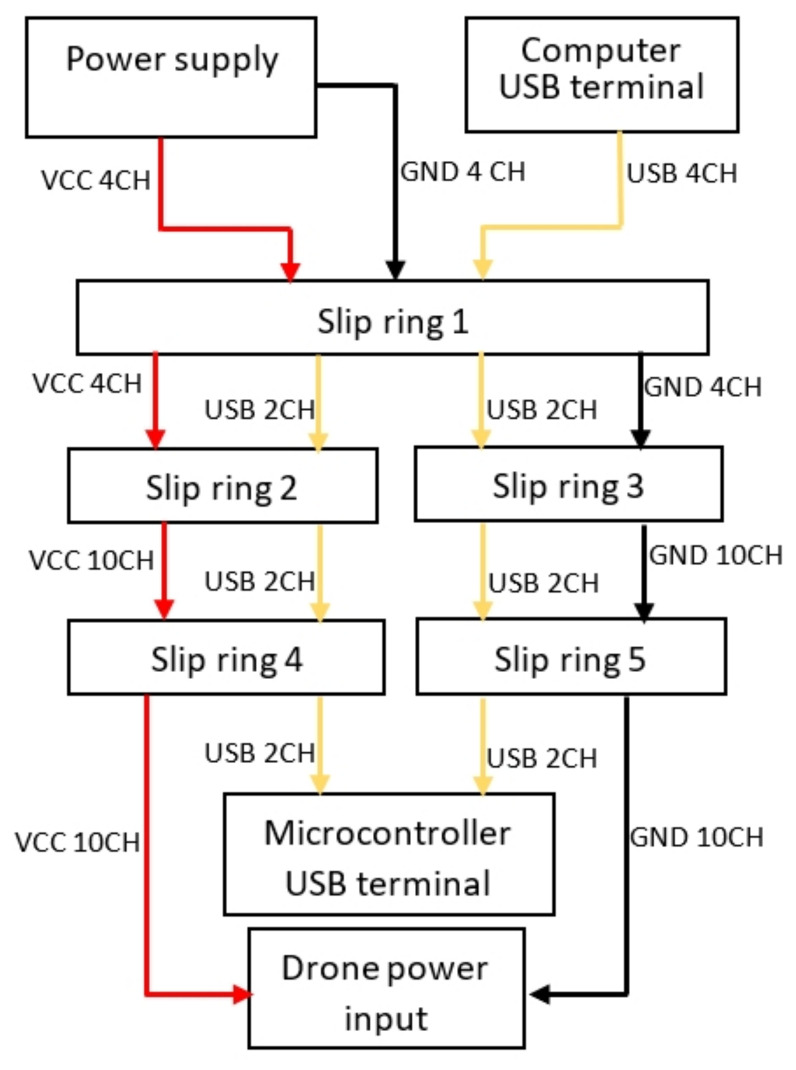
Test platform electrical connection diagram.

As a secondary function, the platform is responsible for conducting electric current from external power supply to vehicle. In addition, it also conducts communication signal between the computer and quadcopter via USB connection. For this purpose, the prototype has integrated connection channels, which are connected via the slip rings. Each ring has 12 connection channels, which are distributed as shown in Figure 8.

Red and black arrows indicate VCC and GND power transmission poles. USB communication is represented by yellow arrows. The number of channels used between each connection is shown next to each arrow.

## 6. Hardware and Software

To implement and execute control algorithms in the prototype, the Pixhawk (PX4) microcontroller is used, which consists of an open hardware autopilot ecosystem based on NuttX operating system (RTOS) with flight control units that are powered by embedded sensors to execute algorithms on ARM^®^ Cortex^®^-M microprocessors, which drive the vehicle’s motors through PWM outputs. The main purpose for employing PX4 hardware in this prototype is to use MathWorks Build Tool Integration (BTI) to enable MATLAB software to invoke the ARM-GCC compiler in building applications based on Simulink block models. The system target file is ert.tlc (Embedded Real-Time) and is available with Embedded Coder.

### 6.1. Capabilities and Features

By using the Embedded Coder ™ support package for PX4^®^ autopilots, it is possible to generate C++ code from Simulink^®^ models designed for Pixhawk FMUs (Flight Management Units). In this context, the PX4 toolchain is used to compile algorithms designed for this flight management unit, in which data from integrated sensors are incorporated [30].

Pixhawk Support Package (PSP) offers the possibility to incorporate Pixhawk Toolchain for compiling and downloading firmware in the Pixhawk FMU unit, which includes a block library for accessing data from inertial sensors, GPS, PWM output, ADC, serial Rx/Tx and other available functions that can be used in Simulink model at runtime. Using this library it is possible to create block models to design a control system to manipulate quadcopter motors. Once this control system is successfully modelled, simulated and verified, PSP generates the source code that is subsequently compiled. Since this tool generates code for a Simulink PX4 module, the Pixhawk support package attaches the generated code to the compilation process to match the general firmware in the built environment when using the CMake command. This source code interfaces between the designed control system and the base hardware drivers that constitute PX4 firmware. As a result of this process, a NuttX application titled PX4_Simlink_App is created with the algorithm defined by the designed block model. This application is included as part of the boot script in the firmware that is compiled and executed in PX4.

As part of the set of PX4 sensors, a magnetometer is integrated, which is used to measure the orientation of the quadcopter with reference to the earth’s magnetic field. As a consequence of electromagnetic interferences existing in a laboratory, interferences generated by motors and current circulating through cables distributed in the prototype, an erroneous data reading is generated by this sensor, causing a deviation in yaw orientation measurement with respect to the actual position. This drawback could be solved by implementing a secondary external magnetometer at a longer distance from motors to reduce electromagnetic interference.

### 6.2. External Mode Execution

External mode is a way in which it is possible to monitor and adjust in real-time the execution of the source code compiled in PX4, from its graphical interface environment. This feature is included with Matlab/Simulink Embedded Coder tool and greatly enhances the interactive debugging capabilities for models executed in real-time by providing all the source files necessary to establish a serial communication channel between the computer and PX4 [31]. Using this mode, the created Simulink model becomes a user interface for interaction with the previously compiled code during code execution, achieving two main actions:

1. Visualize the output value of a given signal at each moment. In addition, it is possible to store signal data and generate a *.mat file.

2. Set parameters as global variables that can be modified in the generated code during its execution, so that an external program can have more control over them in relation to the predefined instruction set.

**Figure 9:**
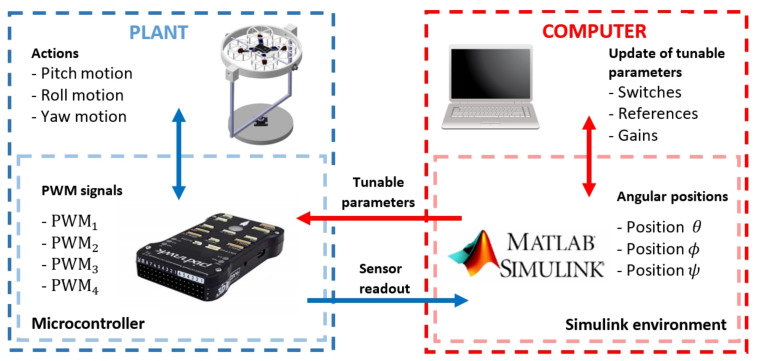
Prototype operation using Matlab Simulink in external mode.

Before starting the simulation of a created application and linking communication between computer and microcontroller, a COM port for USB connection to PX4, a baud rate of 115,200 baud and a timeout of 0.5 s are set. Figure 9 depicts the operation of the prototype with Matlab-Simulink software in external mode while the simulation is running.

Blue box represents the plant, which is constituted by the quadcopter mounted on the test platform. Inside is another box representing PX4 hardware, which controls the platform with the previously compiled algorithm. Both subsystems are in constant feedback through the control algorithm, on the one hand, the microcontroller sends the PWM signal to each motor and on the other hand, the platform performs actions that are subsequently measured by sensors integrated into the microcontroller. The sensed measurements are sent to the computer through the USB connection for storage. Red box represents the computer with Matlab-Simulink environment, where the block diagram of the control system compiled on the Pixhawk FMU board is located. This block system contains a set of adjustable parameters that the user can modify and send to the microcontroller to update the control algorithm that is running. With this set of adjustable parameters, it is possible to set reference signals, gains, switches for control blocks, etc.

## 7. System Parameters Identification

This section sets out parameters that are subsequently used to design a control system. Using a digital scale, the weights of each component of the prototype were measured. From this information, a CAD model was created by making a mass distribution. In this way, the moments of inertia of the prototype were obtained, which are part of the mathematical equations used to model the system. The results obtained from this calculation are as follows: moment of inertia $I_{xx}=0.11$ kgm^2^, moment of inertia $I_{yy}=0.41$ kgm^2^ and moment of inertia $I_{zz}=1.07$ kgm^2^. Other parameters obtained from physical measurements include: total vehicle mass (m)=0.720 kg, arm distance (l)=0.129 m, gravitational acceleration (g)=9.81 m/s^2^.

**Figure 10:**
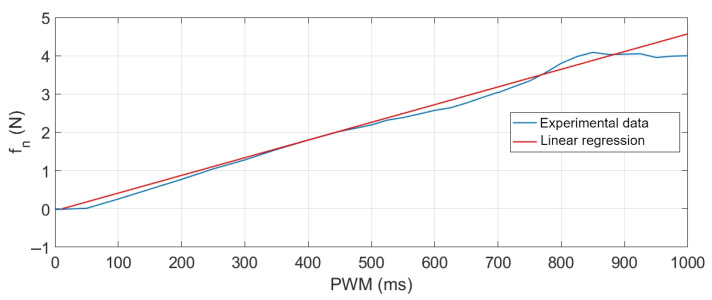
Experimental thrust curve for Emax brushless motor RD2205-2300kV.

Using the RC Benchmark 1580 series dynamometer [32], the thrust produced $f_{n}$ by one of the Emax brushless motors under a PWM signal variation was measured. This test was carried out using propellers model 4045 with three blades. Data from this experiment are shown in Figure 10, where the thrust produced is expressed in Newtons (N) and the period of pulse width modulation in milliseconds (ms). Subsequently, a linear regression was applied to these data (red curve) to finally obtain the function presented in (6), which characterises the force $f_{n}$ produced by each motor at a given PWM signal.

(6) $$PWM=216.49f_{n}+10.06$$

## 8. Model and Control Strategy

In recent years, different mathematical models have been proposed that represent the quadcopter as a sub-actuated mechanical system with six degrees of freedom and only four control inputs. Most authors consider the vehicle as a rigid body, as shown in [33,34]. In a large number of these papers, the controller is developed through a linearized system [35] or by neglecting insignificant terms.

### 8.1. Nonlinear State Space Model

The quadrocopter rotation system represents three degrees of freedom corresponding to angular displacement on its three rotation axes. The dynamic model for this system [36] is indicated by the following differential equations.

$$\dot{\phi }=p+q\sin\phi \tan\theta +r\cos\phi \tan\theta$$

$$\dot{\theta }=q\cos\phi -r\sin\phi$$

$$\dot{\psi }=q\sin\phi \sec\theta +r\cos\phi \sec\theta$$

$$\dot{p}=\frac{I_{yy}-I_{zz}}{I_{xx}}qr+\frac{\tau_{\phi }}{I_{xx}}+\frac{\tau_{\phi_{d}}}{I_{xx}}$$

$$\dot{q}=\frac{I_{zz}-I_{xx}}{I_{yy}}pr+\frac{\tau_{\theta }}{I_{yy}}+\frac{\tau_{\theta_{d}}}{I_{yy}}$$

$$\dot{r}=\frac{I_{xx}-I_{yy}}{I_{zz}}pq+\frac{\tau_{\psi }}{I_{zz}}+\frac{\tau_{\psi_{d}}}{I_{zz}}$$

These scalar equations of motion describe the nonlinear dynamics of vehicle, taking a symmetric rigid body approach subjected to external forces applied at its center of mass. Where [ϕ,θ,ψ] and [p,q,r] denote angular positions and velocities respectively shown in Figure 3, [$I_{xx}$,$I_{yy}$, $I_{zz}$] represent the vehicle moments of inertia, [$\tau_{\phi },\tau_{\theta },\tau_{\psi }$] are the torques from Equations (2), (4) and (5) that induce angular motion in quadcopter. [$\tau_{\phi_{d}},\tau_{\theta_{d}},\tau_{\psi_{d}}$] represent gyroscopic and aerodynamic disturbance effects.

### 8.2. Linear State Space Model

In order to focus the analysis to a specific operating point of interest, linearization of system is carried out. Using equations that describe the nonlinear model, the linearized system defined by the Equations (7)–(12) is obtained:

(7) $$\dot{\phi }=p$$

(8) $$\dot{\theta }=q$$

(9) $$\dot{\psi }=r$$

(10) $$\dot{p}=\frac{\tau_{\phi }}{I_{xx}}$$

(11) $$\dot{q}=\frac{\tau_{\theta }}{I_{yy}}$$

(12) $$\dot{r}=\frac{\tau_{\psi }}{I_{zz}}$$

where the equilibrium point selected for linearization refers to the stationary flight of aircraft, where all moments are counteracted and the net thrust force is equivalent to vehicle weight.

As a consequence of an insufficient thrust generation for this particular quadcopter model, there is a very small operating margin to manipulate yaw movements. In addition to this problem, there is also the previously described problem of yaw angle measurement error caused by electromagnetic interference in the magnetometer. Due to these factors, it makes no sense to design a control system to operate with yaw motion, so it is decided to restrict this movement and design a control system for pitch and roll motions.

### 8.3. Linear State-Space Model for Pitch and Roll Motions

As discussed at the end of the previous section, the control design is focused on the pitch and roll dynamics. For that reason, the linearized three DoF model described by Equations (7)–(12) is reduced to two DoF model.

As a result of the linearization process, a complete decoupled model is obtained, so that each rotational motion is expressed independently. This approach is appropriate when the system operates around the selected equilibrium point, then the effect between motions is negligible. From this approach, Equations (9) and (12) belonging to yaw motion are dispensed with and the remaining equations are maintained.

(13) $$\left\{\begin{array}{c} \dot{x}\left(t\right)=Ax+Bu\\ y(t)=Cx \end{array}\right.$$

So, by performing a matrix approach of Equations (7), (8), (10) and (11) in form (13), the system is defined by the matrices below:

$$A=\left(\begin{array}{cccc} 0 & 1 & 0 & 0\\ 0 & 0 & 0 & 0\\ 0 & 0 & 0 & 1\\ 0 & 0 & 0 & 0 \end{array}\right)\;;\;B=\left(\begin{array}{cc} 0 & 0\\ \frac{1}{Ixx} & 0\\ 0 & 0\\ 0 & \frac{1}{Iyy} \end{array}\right)\;;\;C=(\begin{array}{cccc} 1 & 0 & 0 & 0\\ 0 & 0 & 1 & 0 \end{array})$$

where *A* is the state matrix of dimension 4×4, *B* is the input matrix of dimension 4×2 and *C* is the output matrix of dimension 2×4. On the other hand, the state vector is constituted as $x=[\phi ,\dot{\phi },\theta ,\dot{\theta }]$, the input vector is composed by $u=[\tau_{\phi },\tau_{\theta }]$ and the output vector is defined as y=[ϕ,θ].

### 8.4. Control Scheme for System Stabilization

**Figure 11:**
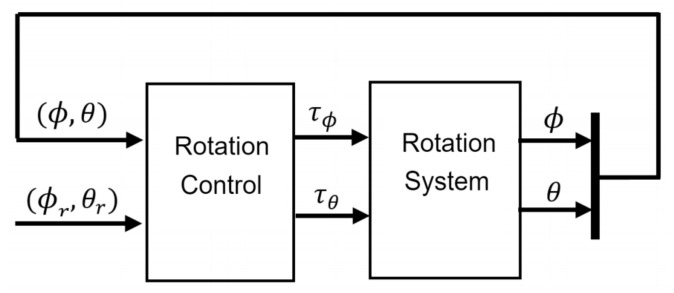
Control scheme for rotation system.

The control approach for stabilization of the tilt angles ϕ and θ is schematized in Figure 11. In this diagram, there are four elements as inputs to the controller, [ϕ,θ] are the two tilt angles of the quadcopter to be controlled and [$\phi_{r},\theta_{r}$] are the reference angles for each system output. The outputs of the controller are torques [$\phi_{r},\theta_{r}$] which are the inputs to the rotation system responsible for generating vehicle actuation. Specifically, the input $\tau_{\phi }$ controls roll motion and $\tau_{\theta }$ controls pitch motion.

**Figure 12:**
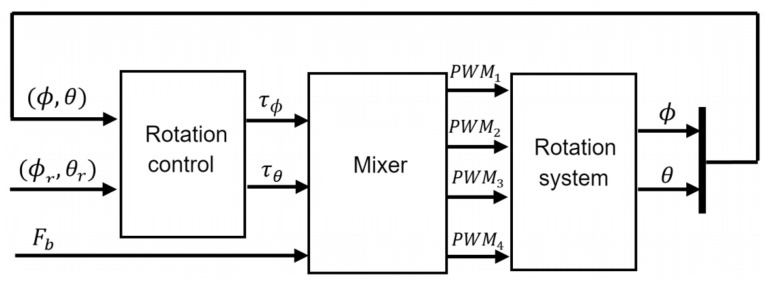
Integration of Mixer block to the control scheme for rotation system.

From this approach, a second one is developed (Figure 12) in which the system inputs are expressed as PWM signals. These are created by mixer block, which is responsible for filtering signal coming from rotation control to PWM signal and distribute to each motor of the quadcopter. The inputs of this block are torques [$F_{b}$,$F_{b}$] resulting from the calculation made by the rotation controller and base on the thrust force $\left(F_{b}\right)$, which is proportionally distributed.

From the torque definition, the set of forces required by each motor to generate torques demanded by the control system are calculated using following equations:

$$F_{\phi }=\tau_{\phi }/4d\;;\;F_{\theta }=\tau_{\theta }/4d$$

where *d* is the parameter defined in Equation (3) and [$F_{\phi }$, $F_{\theta }$] are forces required to generate pitch and roll torques respectively. From calculation obtained by these equations, (6) is used to obtain PWM signal modulation, defining $PWM_{\phi }$ and $PWM_{\theta }$ signals associated to each movement. Subsequently, Equations (14)–(17) distribute the signal to each motor so that together they contribute in equal proportion to required torques. Where $PWM_{1},PWM_{2},PWM_{3},PWM_{4}$ are the signals sent to the motors 1, 2, 3 and 4 respectively.

(14) $$PWM_{1}=PWM_{\phi }+PWM_{\theta }+PWM_{b}+1000$$

(15) $$PWM_{2}=-PWM_{\phi }+PWM_{\theta }+PWM_{b}+1000$$

(16) $$PWM_{3}=-PWM_{\phi }-PWM_{\theta }+PWM_{b}+1000$$

(17) $$PWM_{4}=PWM_{\phi }-PWM_{\theta }+PWM_{b}+1000$$

In addition, in these expressions is also added the base thrust $F_{b}$ with which the system operates. This is added in PWM signal form ($PWM_{b}$). The magnitude of this force is set by the user at each point in time. Constant 1000 is set so that motors start receiving a PWM signal magnitude from a suitable range to start accelerating.

## 9. Controller Development

In order to show the potential of the platform for implementation, experimentation and evaluation of a control system, it is proposed a practical exercise to design a control law using two techniques well studied, PID from the classical control theory and state feedback from the modern control theory.

### 9.1. Basic Control Theory Student Lab Assignment: PID-Based Controller Design

As mentioned at beginning of Section 8.3, the set of equations shown in (7)–(12) describes a fully decoupled rotating system. The matrix representation for pitch and roll motions according to these equations is as follows:

**Subsystem for roll motion**

$$A_{\phi }=\left(\begin{array}{cc} 0 & 1\\ 0 & 0 \end{array}\right)\;;\;B_{\phi }=\left(\begin{array}{c} 0\\ 1/I_{xx} \end{array}\right)\;;\;C_{\phi }=\left(\begin{array}{cc} 1 & 0 \end{array}\right)$$

where the input is $u_{\phi }=\tau_{\phi }$ and the output is $y_{\phi }=\phi$.

**Subsystem for pitch motion**

$$A_{\theta }=\left(\begin{array}{cc} 0 & 1\\ 0 & 0 \end{array}\right)\;;\;B_{\theta }=\left(\begin{array}{c} 0\\ 1/I_{yy} \end{array}\right)\;;\;C_{\theta }=\left(\begin{array}{cc} 1 & 0 \end{array}\right)$$

where the input is $u_{\theta }=\tau_{\theta }$ and the output is $y_{\theta }=\theta$.

As can be seen, each movement is a function of single control input. Subsequently, it is possible to go from state-space to transfer function representation using Equation (18).

(18) $$G\left(s\right)=C{\left(sI-A\right)}^{-1}B$$

The transfer functions are expressed as $G{\left(s\right)}_{\phi }$ y $G{\left(s\right)}_{\theta }$ for each subsystem as follows:

$$G{\left(s\right)}_{\phi }=\frac{1}{s^{2}I_{xx}}\;;\;G{\left(s\right)}_{\theta }=\frac{1}{s^{2}I_{yy}}$$

**Figure 13:**
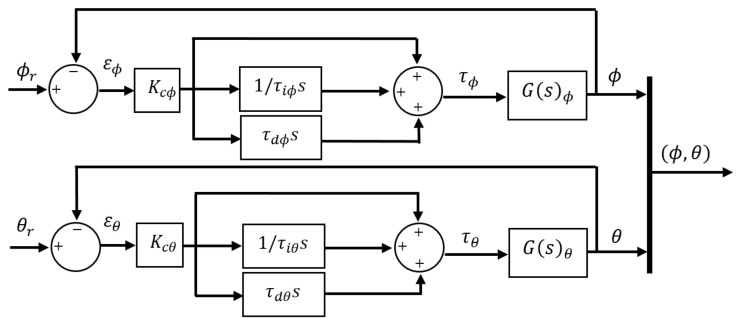
PID control approach with ISA structure.

Once model is defined in this form, it is easy to define the closed loop for each system output. Figure 13 shows the block diagram that refers to PID control approach with ISA structure, where it is identified [$K_{c_{\phi }},\tau_{i_{\phi }},\tau_{i_{\phi }}$] as the control action coefficients for roll motion and [$K_{c_{\theta }},\tau_{i_{\theta }},\tau_{d_{\theta }}$] for pitch motion.

Therefore, each controller is defined by the functions $C{\left(s\right)}_{\phi }$ and $C{\left(s\right)}_{\theta }$ as follows:

$$C{\left(s\right)}_{\phi }=K_{c_{\phi }}\left(1+\frac{1}{\tau_{i_{\phi }}s}+\tau_{d_{\phi }}s\right)$$

$$C{\left(s\right)}_{\theta }=K_{c_{\theta }}\left(1+\frac{1}{\tau_{i_{\theta }}s}+\tau_{d_{\theta }}s\right)$$

Due to the unstable nature of quadcopters and the difficulty of performing open-loop experiments, it is difficult to establish a set of dynamic specifications that would be appropriate for the controllers that the students will design. For that reason, a previous state-space feedback control law has been obtained via Linear Matrix Inequalities (LMI) [37]. In particular, the stability problem has been formulated and solved in terms of LMIs, where parametric uncertainties in the moments of inertia present in the model shown in (7)–(12) have been considered under the operating ranges shown in Table 4.

As mentioned before, the purpose of this control analysis is to define a set of appropriate dynamic specifications for the students. The resolution of this approach leads to the following dynamic specifications:

- Roll motion ϕ: $T_{ss}\approx 8s$, $M_{p}\approx 40\%$, ς≈0.28 and $\omega_{n}\approx 1.32$.
- Pitch motion θ: $T_{ss}\approx 4.s$, $M_{p}\approx 9\%$, ς≈0.6 and $\omega_{n}\approx 1.22$.

Where $T_{ss}$ is the settling time of the system response, $M_{p}$ is the maximum overshoot, $\omega_{n}$ is the natural frequency of the system and ς is the damping factor. These specifications define the closed-loop poles $\omega_{p\phi }$ for roll motion and $\omega_{p\theta }$ for pitch motion which are shown below:

$$\Omega_{p\phi }=\left\{-0.3700\pm 1.2666i;-0.4728\right\}$$

$$\Omega_{p\theta }=\left\{-0.7467\pm 0.9726i;-0.1348\right\}$$

For the design of the PID gains for pitch and roll control, it is proposed to use the technique based on pole placement [38], where a reference close-loop behaviour is described by $M\left(s\right)=\frac{K\omega_{n}^{2}}{s^{2}+2\varsigma \omega_{n}s+\omega_{n}^{2}}$. PID control parameters with the structure $C\left(s\right)=K_{c}\left(1+\frac{1}{\tau_{i}s}+\tau_{d}s\right)$ are obtained by solving the close-loop equation $M\left(s\right)=\frac{C(s)G(s)}{1+C(s)G(s)}$. Following this approach, the parameters for each PID controller are the following:

$$K_{c_{\phi }}=0.230;\tau_{i_{\phi }}=2.540;\tau_{d_{\phi }}=0.580$$

$$K_{c_{\theta }}=0.830;\tau_{i_{\theta }}=3.860;\tau_{d_{\theta }}=0.910$$

### 9.2. Advanced Control Theory Student Lab Assignment: Controller Design from a State Feedback

**Figure 14:**
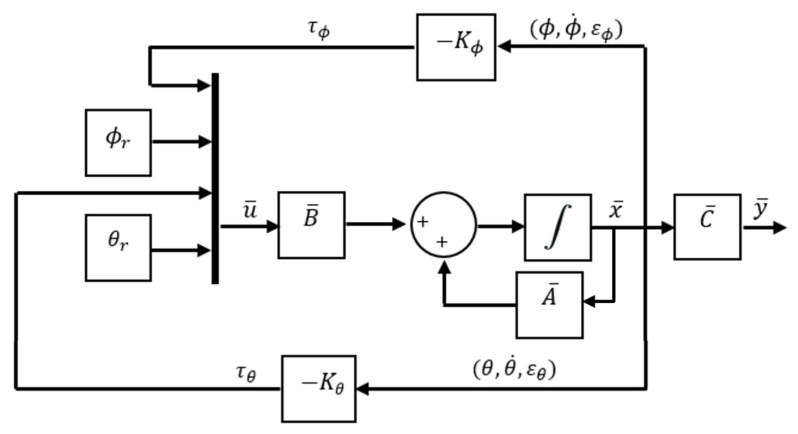
State feedback control approach.

To establish the control model under this approach and in order to ensure the system reaches a given reference, an integrator is inserted between the error estimator and the plant by formulating an extra equation for each output in the control model. These equations are:

$${\dot{\varepsilon }}_{\phi }=\phi_{r}-\phi \;;\;{\dot{\varepsilon }}_{\theta }=\theta_{r}-\theta$$

where [$\phi_{r}$, $\theta_{r}$] represent reference signals imposed for each output and [${\dot{\varepsilon }}_{\phi }$, ${\dot{\varepsilon }}_{\theta }$] symbolize error dynamics through its derivative for each output. Adding this system of equations to model presented in Section 8.3 yields the model detailed in (19).

(19) $$\begin{array}{c} \bar{A}=\left(\begin{array}{cccccc} 0 & 1 & 0 & 0 & 0 & 0\\ 0 & 0 & 0 & 0 & 0 & 0\\ -1 & 0 & 0 & 0 & 0 & 0\\ 0 & 0 & 0 & 0 & 1 & 0\\ 0 & 0 & 0 & 0 & 0 & 0\\ 0 & 0 & 0 & -1 & 0 & 0 \end{array}\right);\bar{B}=\left(\begin{array}{cccc} 0 & 0 & 0 & 0\\ \frac{1}{Ixx} & 0 & 0 & 0\\ 0 & 1 & 0 & 0\\ 0 & 0 & 0 & 0\\ 0 & 0 & \frac{1}{Iyy} & 0\\ 0 & 0 & 0 & 1 \end{array}\right);\\ \bar{C}=\left(\begin{array}{cccc} 1 & 0 & 0 & 0\\ 0 & 0 & 0 & 1 \end{array}\right) \end{array}$$

where $\bar{A}$, $\bar{B}$ and $\bar{C}$ are augmented matrices of dimension 6×6, 6×4 and 2×4 respectively, the state vector is constituted by $\bar{x}=\left[\phi ,\varepsilon_{\phi },\theta ,\dot{\theta },\varepsilon_{\theta }\right]$, the augmented input vector is composed by $\bar{u}=$[$\tau_{\phi }$,$\phi_{r}$,$\tau_{\theta }$,$\theta_{r}$] and the output vector is defined as $\bar{y}\;=\;$[ϕ,θ]. The closed-loop scheme for state feedback is presented in Figure 14.

(20) $$\tau_{\phi }=-\left(k_{\phi_{1}}\phi +k_{\phi_{2}}\dot{\phi }\right)+k_{\phi_{3}}\varepsilon_{\phi }$$

(21) $$\tau_{\theta }=-\left(k_{\theta_{1}}\theta +k_{\theta_{2}}\dot{\theta }\right)+k_{\theta_{3}}\varepsilon_{\theta }$$

where vector $K_{\phi }$ is composed by elements [$k_{\phi_{1}}$,$k_{\phi_{2}}$,$-k_{\phi_{3}}$] and vector $K_{\theta }$ is constituted by [$k_{\theta_{1}}$,$k_{\theta_{2}}$,$-k_{\theta_{3}}$]. Making use of the control law u=−Kx, the controller for this model is defined by Equations (20) and (21). Where $k_{\phi_{1}}$, $k_{\phi_{2}}$, $k_{\phi_{3}}$, $k_{\theta_{1}}$, $k_{\theta_{2}}$, $k_{\theta_{3}}$ are feedback constants defined by eigenvalue reassignment.

To design steady-state feedback, it is essential to verify system controllability. From evaluating the controllability matrix derived from the system defined by matrices in (19) it is possible to infer that it is a fully controllable system of rank 6 and is characterised by the following open-loop eigenvalue set: $\Omega_{A}=\left\{0,0,0,0,0,0\right\}$.

Then, to create the controller it is proposed to set the closed-loop poles $\omega_{p\phi }$ and $\omega_{p\theta }$ to match the eigenvalues assigned in state feedback approach. So, once the system controllability is verified, the feedback state matrix *K* is calculated by using the vector [$\omega_{p\phi }$,$\omega_{p\theta }$] and the eingevalue placement method by the Ackermann function implemented in Matlab [39]. The result is as follows:

$$K=\left(\begin{array}{cccccc} 0.230 & 0.123 & 0.090 & 0 & 0 & 0\\ 0 & 0 & 0 & 0 & 0 & 0\\ 0 & 0 & 0 & 0.830 & 0.750 & 0.210\\ 0 & 0 & 0 & 0 & 0 & 0 \end{array}\right)$$

Using the gain values obtained in *K*, the control law for each system input is presented in (22) and (23).

(22) $$\tau_{\phi }=-\left(0.230\phi +0.123\dot{\phi }\right)+0.090\varepsilon_{\phi }$$

(23) $$\tau_{\theta }=-\left(0.830\theta +0.750\dot{\theta }\right)+0.210\varepsilon_{\theta }$$

## 10. Implementation and Results

As a prototype focused on educational environments, it is of special interest to highlight the level of practicality and simplicity of the platform to carry out implementation and evaluation of designed controllers. As described in Section 6, the main interaction tool between user and platform is Matlab-Simulink, where block diagrams for control systems are edited. In addition, as auxiliary functions provided by this tool, it is possible to modify system parameters, enter commands to execute predefined actions and monitor both control inputs and system response in real-time.

Experimental results obtained with the implementation of controllers developed in Section 9.1 and Section 9.2 are presented below.

### 10.1. State Feedback Control Experimentation

**Figure 15:**
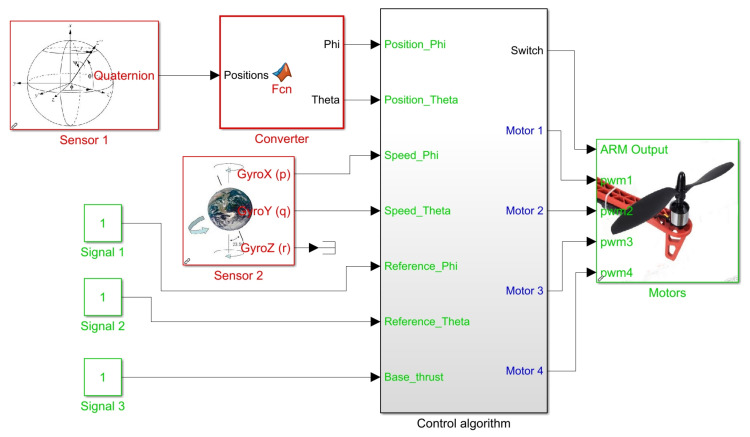
Simulink block diagram for state feedback controller implementation.

According to the scheme shown in Figure 14, continuous measurement of the elements in the state vector $\bar{x}$ is required to feed back this control loop. Figure 15 presents the Simulink block system created to implement this controller. Blocks highlighted in red allow reading angular positions and velocities received from sensors, while blocks in green “Signal 1”, “Signal 2” and “Signal 3” are set by user at runtime to send reference signals [$\phi_{r}$,$\theta_{r}$] and to set the base thrust signal $PWM_{b}$ present in (14)–(17).

**Figure 16:**
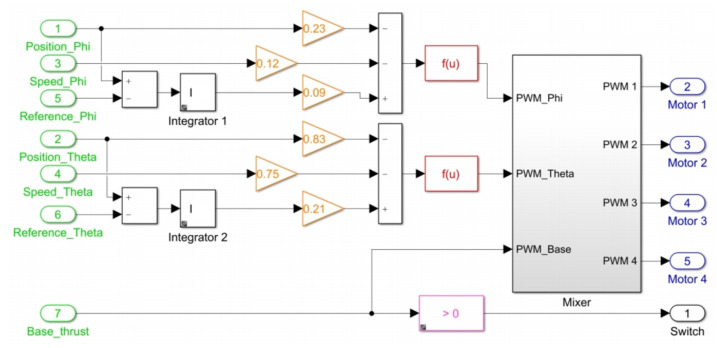
Block diagram in “Control algorithm” subsystem for state feedback controller implementation.

The subsystem entitled “Control Algorithm” processes all these inputs to define as a result of a PWM signal to each motor of quadcopter at each time in the block titled “Motors”. Figure 16 illustrates the internal block diagram in this subsystem.

Green blocks with numbers 1–4 represent states contained in vector $\bar{x}$ which refer to angular positions and velocities, blocks 5 and 6 are the reference signals applied to calculate the errors [$\varepsilon_{\phi },\varepsilon_{\theta }$] which constitute the 2 remaining states in $\bar{x}$. Block number 7 is the base thrust set. Orange blocks are gains of the control laws defined in (22)–(23) for computation of stabilization torques [$\tau_{\phi }$,$\tau_{\theta }$]. Red blocks are functions that evaluate the magnitude of each torque and define a required thrust force in PWM signal using Equation (6). The “Mixer” subsystem handles the proportional signal distribution for each motor using Equations (14)–(17). The pink block acts as a switch for disarming the motors when zero base thrust is set.

**Figure 17:**
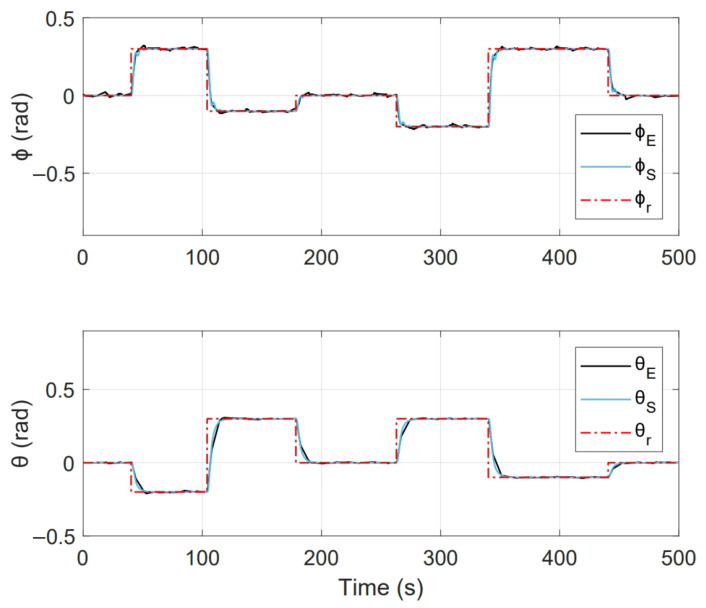
System response obtained in state feedback control experiment.

Figure 17 shows the system response of an experiment done with this implementation, where initial conditions [$\phi_{0},\theta_{0}$] = [0,0] and duration 500 s. Where [$\phi_{r},\theta_{r}$] represent the imposed reference signals, [$\phi_{E},\theta_{E}$] are the system response on the test platform and [$\phi_{S},\theta_{S}$] are the numerical simulation response.

**Figure 18:**
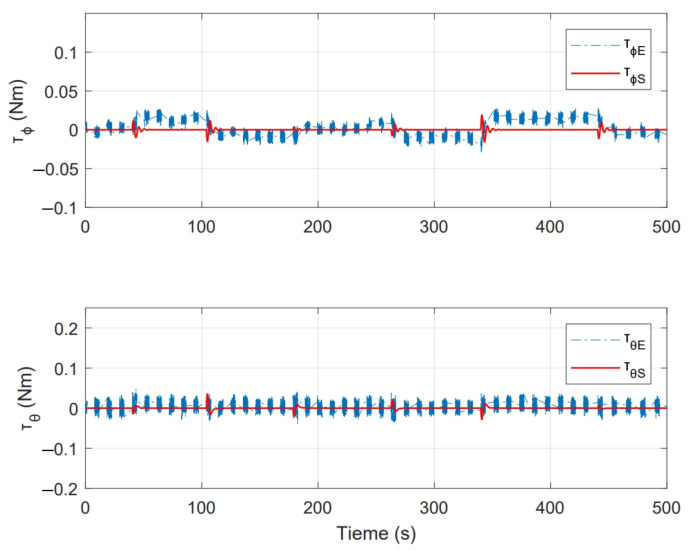
Control inputs presented in state feedback experiment.

On the other hand, Figure 18 shows the control actions applied, where [$\tau_{\phi_{E}},\tau_{\theta_{E}}$] are the stabilisation torques calculated by the controller in experimental test and [$\tau_{\phi_{S}},\tau_{\theta_{S}}$] are the control actions calculated during numerical simulation.

**Figure 19:**
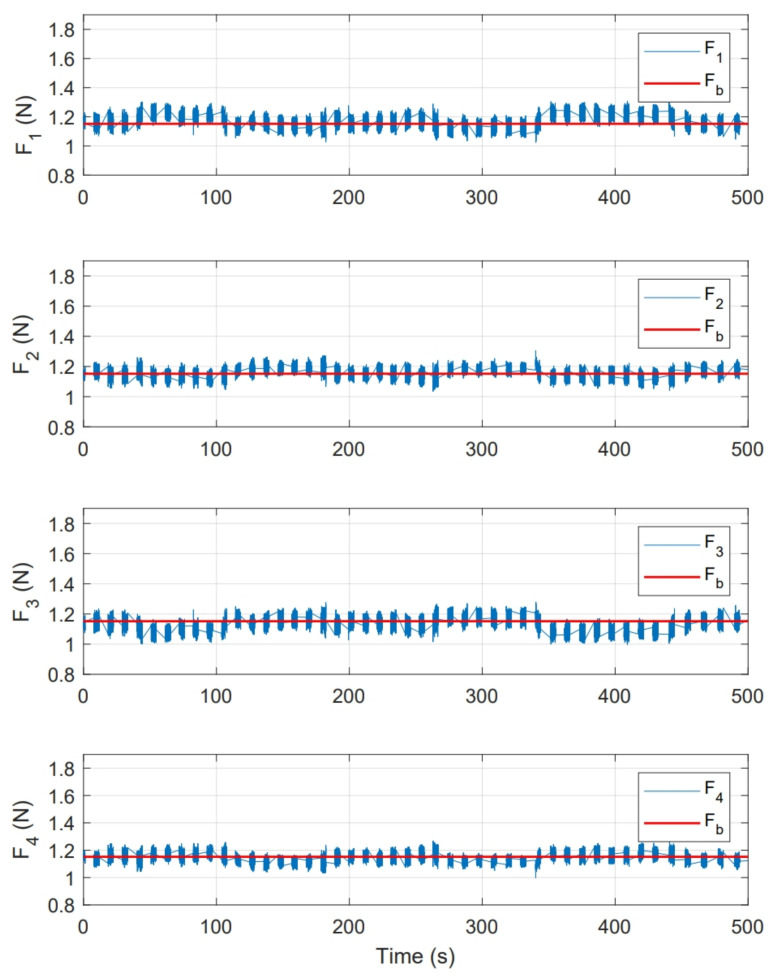
Thrust force presented for each motor in state feedback experiment.

Figure 19 shows the magnitude of thrust force for each motor $f_{1}$, $f_{2}$ ,$f_{3}$ and $f_{4}$. This figure also shows the magnitude of base force $F_{b}$ used in whole experiment, which corresponds to $PWM_{b}$ signal established in Equations (14)–(17), around this force, compensations computed by controller are applied.

### 10.2. PID Control Results

**Figure 20:**
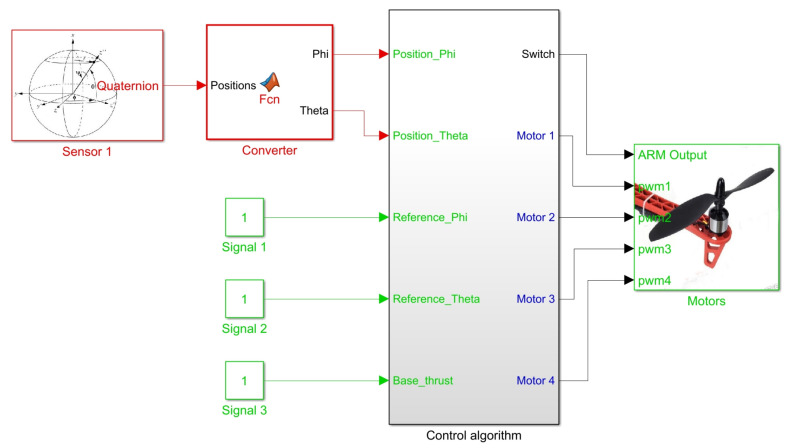
Simulink block diagram for PID controller implementation.

**Figure 21:**
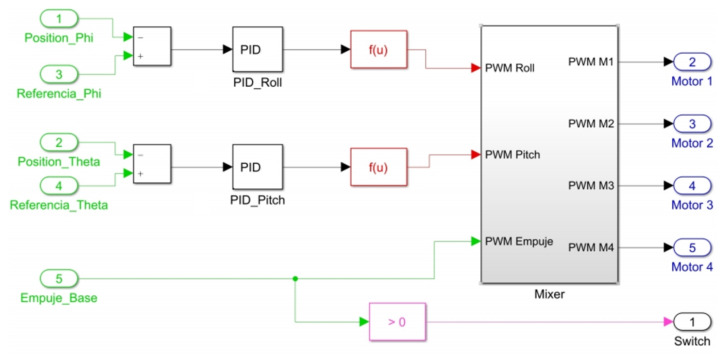
Block diagram in “Control algorithm” subsystem for PID controller implementation.

To create the block diagram for this approach, a similar structure to the one shown in Figure 15 is used, but with the difference that the control loop is fed back only with the information provided by the errors between angular positions and reference signals. Figure 20 shows the block diagram to implement PID controller. The function of the blocks with similar titles to those presented in Figure 15 is the same. The subsystem with the title “Control Algorithm” contains the internal PID control system to carry out control actions. This subsystem is shown in Figure 21, where green blocks with numbers 1–4 define the errors [$\varepsilon_{\phi },\varepsilon_{\theta }$] in which control actions act for feedback. The PID(z)s blocks represent these control actions, which are created from structure and gains presented in Section 9.2.

In addition, an anti-windup mechanism is added in each loop to saturate each torque to a limit of 0.8 Nm. The blocks in common in this subsystem with those in Figure 16 have the same function as described before.

**Figure 22:**
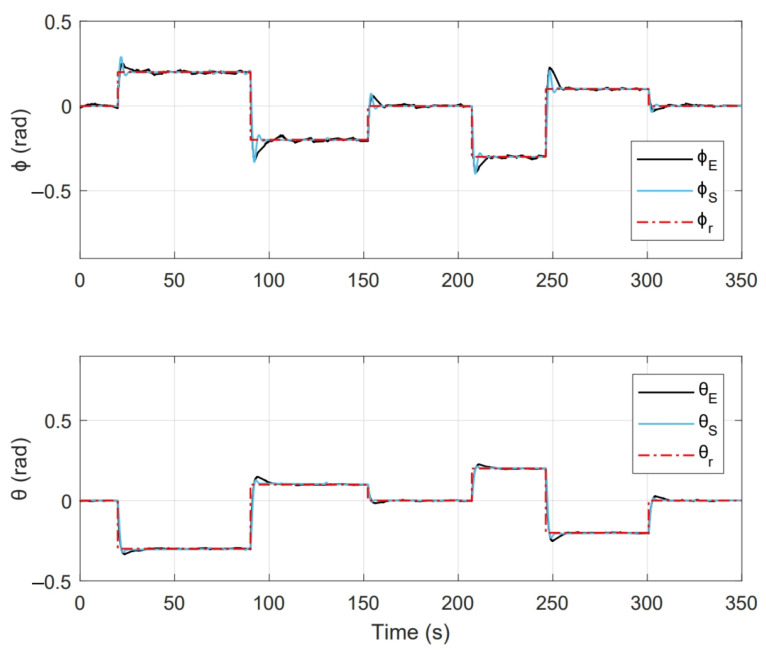
System response obtained in PID control experiment.

**Figure 23:**
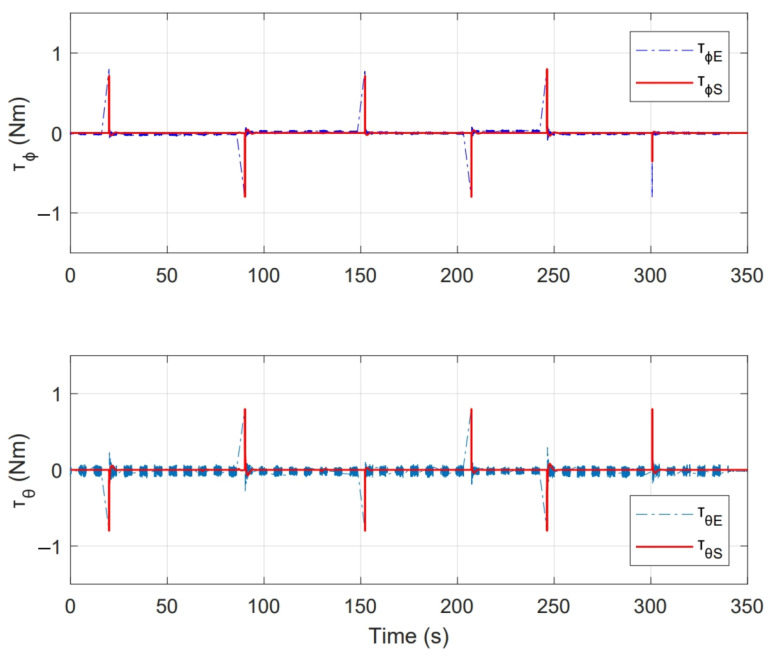
Control inputs presented in PID control experiment.

**Figure 24:**
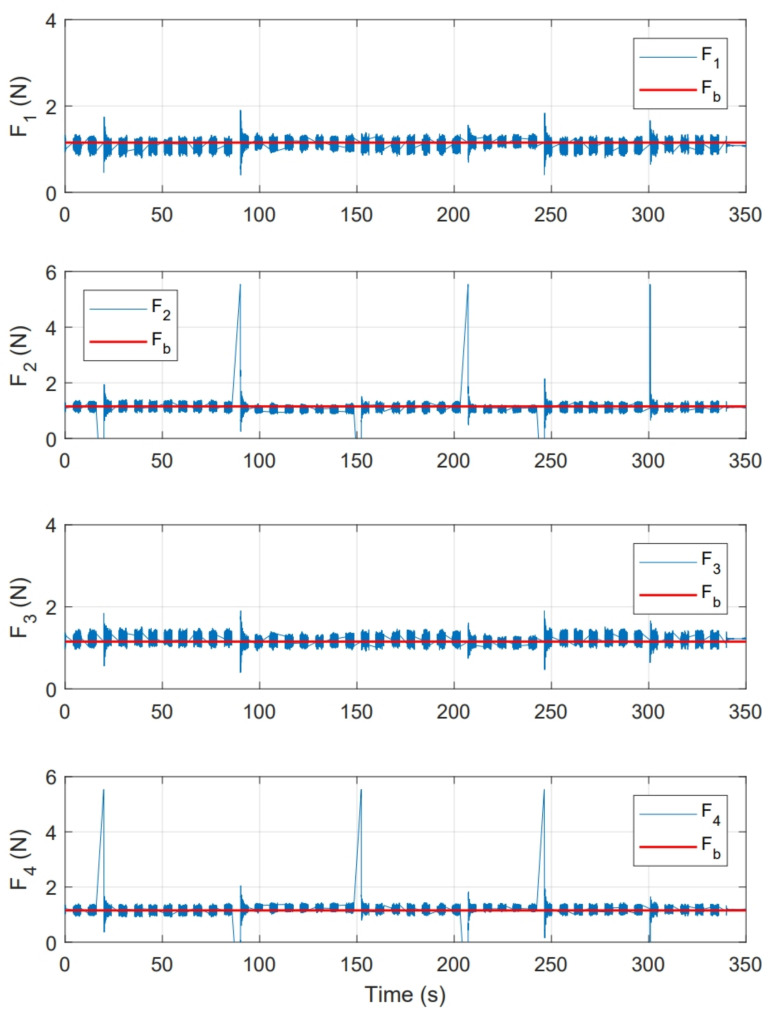
Thrust force presented for each motor in PID control experiment.

As a second test, an experiment was performed for a period of 350s. Initial conditions were [$\phi_{0}$,$\theta_{0}$] = [0,0]. Figure 22 shows the system response in this second test, where [$\phi_{r},\theta_{r}$] are the imposed reference signals, [$\phi_{E},\theta_{E}$] represent the system response and [$\phi_{S},\theta_{S}$] the system response in numerical simulation. On the other hand, Figure 23 shows the control inputs to carry out each movement, where [$\tau_{\phi_{E}},\tau_{\theta_{E}}$] are inputs present in real test and [$\tau_{\phi_{S}},\tau_{\theta_{S}}$] are control actions calculated in numerical simulation. Figure 24 illustrates thrust forces $f_{1}$, $f_{2}$, $f_{3}$ and $f_{4}$ respectively for each motor in this experiment.

One of the main differences between the experimental and numerical response in Figure 18, Figure 19, Figure 23 and Figure 24 for the experiments carried out is the offset observed between $\tau_{\phi_{E}}$ and $\tau_{\phi_{S}}$. This is related to the additional mechanical resistance added to the quadcopter system due to the platform connections that support the vehicle. This factor limits the operation of the controller for the compensation of very small errors. The same offset is found between $\tau_{\theta_{E}}$ and $\tau_{\theta_{S}}$, although on a smaller magnitude. This indicates that there is a higher mechanical resistance in the operation of roll motion in relation to pitch motion. In any case, the assumption of these drawbacks does not seem to significantly affect the system response in Figure 17 and Figure 22, since the system reaches the references effectively during the experiments. One more point to discuss is the periodic interruption in signal transmission due to the speed difference between microcontroller and computer processing. However, this data interruption is only perceived in the data displayed on the computer and does not represent a problem for the control system compiled in the microcontroller, since as observed in the scheme of Figure 9 it has its own feedback.

Finally, the .slx files with the Simulink block diagrams used in this section to carry out experiments can be found as application examples in the documentation folder for this prototype, which is accessible in [28].

## 11. Conclusions and Future Work

This work has presented the design of a test platform with a gyroscopic structure. Its main purpose is to validate control systems for quadcopters. It is considered a useful prototype to evaluate the performance and operation of control systems, minimizing the necessity for an experienced pilot to operate an aerial vehicle. Due to the manufacturing and assembly processes involved in its construction, it is easy to replicate this prototype at a lower cost compared to existing platforms on the market. Due to its unlimited autonomy and safety level, it can be used in a university laboratory for an indefinite time. The integration of Matlab/Simulink as user interface facilitates its adaptation to the student environment. This tool represents the work area where students can formulate their own control algorithms by creating diagrams using the Pixhawk block library to access sensor data and drive motors. In this context, editing or creating a new block diagram to modify or define a control algorithm to experiment on the platform becomes a very simple process.

Since the main focus of this paper is the presentation of the test platform, the description of its design, manufacture, application and main features, the control approaches presented here have been kept as simple as possible, in order to show as simple and clear as possible the process of implementation and experimentation of a control system without diverting the main focus of the paper to the development of more advanced control techniques. In this sense, the evaluation of a more sophisticated control system design for optimal performance of the platform is established as future work or as part of the assignments to be carried out by students in robotics and automatic control laboratories.

In addition to the laboratory assignments already described above, some application goals such as control design for angular position under static or dynamic loads, automatization of motions for trajectory tracking, and development of protocols for physical failures in the system (e.g., loss of thrust efficiency in a motor) are also proposed. In the field of construction design, it is proposed to use this platform as a test bench to analyse vehicle performance when implementing different components (experiment with propellers and motors of different specifications, evaluate the accuracy of sensors, etc.). In the field of teaching, this platform can also be used as a functional model for a fundamental understanding of quadcopter dynamics or pilot training (e.g., to practice complicated acrobatic aerial manoeuvres).

Finally, as future work, two possible modifications to the prototype are proposed in order to improve performance and solve the current drawbacks.

1. Add encoders on the three axes of rotation that validate/contrast the IMU’s measurement of Euler angles and, at the same time, deal with the signal problem in yaw angle estimation.

2. Analyze possible design modifications to the qav250 quadcopter frame to improve maneuverability in all axes, including yaw motion.

## Figures and Tables

**Table 1:** Desired background knowledge for students.

Background Knowledge	Level
C/C++ programming skills	Basic
MATLAB/Simulink	Medium
Signal processing	Basic
Computer skills	Basic
Control theory	Basic/Medium
Quadcopter dynamics	Basic
Electronics	Basic
Design and assembly of multirotors	Basic
Safety in robotic laboratories	Basic

**Table 2:** Feature comparison between existing test platforms for quadcopters.

	PT-1	PT-2	PT-3	PT-4
Degrees of freedom	3	3	3	3
System autonomy	Unlimited	Unlimited	Limited	Unlimited
Software	Matlab	Matlab	Private	Private
Cost	Low	Low	High	High
Security Level	High	Medium	Medium	High
Pitch range	360°	360°	360°	75°
Roll range	360°	360°	360°	75°
Yaw range	360°	360°	360°	360°
Experimental flexibility	High	Low	Low	Low
Replication level	High	High	Low	Low

**Table 3:** Electronic components specifications.

Component	Brand	Model
Brushless motors	Emax	RD2205-2300
Speed controls	ZTW	Beatles 30A BEC
Energy Distribution Card	REALACC	XT60
Microcontroller	3DR	Pixhawk 1 PX4
Power supply	Mean Well	RS-150-12

**Table 4:** Ranges of uncertainties considered in the moments of inertia of the system.

Moment of Inertia	Nominal	Upper Limit	Lower Limit
$I_{xx}$	0.26 Kgm^2^	0.312 Kgm^2^	0.208 Kgm^2^
$I_{yy}$	0.44 Kgm^2^	0.528 Kgm^2^	0.352 Kgm^2^

## Data Availability

Not applicable.
